# Development of the Wheelchair Interface Questionnaire and initial face and content validity

**DOI:** 10.4102/ajod.v8i0.520

**Published:** 2019-03-28

**Authors:** Karen Rispin, Abigail B. Davis, Vicki L. Sheafer, Joy Wee

**Affiliations:** 1 Department of Biology and Kinesiology, LeTourneau University, Longview, United States; 2 Department of Psychology, LeTourneau University, Longview, United States; 3 Canadian Association of Physical Medicine and Rehabilitation, Kingston, Canada

## Abstract

**Background:**

Because resources are limited in low- and middle-income countries (LMIC), the development of outcome measures is of interest. Wheelchair outcome measures are useful to support evidence-based practice in wheelchair provision.

**Objectives:**

The Wheelchair Interface Questionnaire (WIQ) is being developed to provide a professional perspective on the quality of the interface between a wheelchair and its user. This article discusses the development of the WIQ and its face and content validity.

**Method:**

During field studies in Kenya, we sought to include professional report data on the wheelchair–user interface that could be analysed to inform design changes. None of the existing measures was focused on the interface between users and their wheelchairs. The WIQ was developed to meet this need. To investigate face and content validity, 24 experienced wheelchair professionals participated in a study that included two rounds of an online survey and a focus group in Kenya.

**Results:**

Responses were categorised by topic and the WIQ was modified following each iteration. Participants affirmed the usefulness of a brief professional report measure to provide a snapshot of the user–wheelchair interface. Participants emphasised the importance of brevity, wide applicability and provision of specific feedback for wheelchair modification or design changes. The focus group agreed that the final version provided useful data and was applicable to virtually all wheelchair users in LMIC.

**Conclusion:**

These preliminary studies indicate initial face and content validity of the WIQ as a method for providing a professional perspective on the interface between a user and his or her wheelchair.

**Keywords:**

Outcome measure; wheelchair assessment; user–wheelchair interface; wheelchair appropriateness; professional report.

## Introduction

It is estimated that about 1% of the global population requires a wheelchair. A large percentage of the people who do not have access to wheelchairs live in low- and middle-income countries (LMIC) (World Health Organization 2011). In addition, many wheelchairs lack adequate durability, do not provide satisfactory facilitation of mobility or are inappropriate for users’ needs and situations (Pearlman 2006; Visagie et al. 2016). Yet access to effective and appropriate wheelchairs has many important health, economic and social benefits for individuals, as well as societal benefits such as productivity and effective use of health resources (Bray et al. 2014; Visagie et al. 2016; World Health Organization 2011). Because of the growing need for wheelchairs and the issue of wheelchair effectiveness, it is critical to provide evidence-based data to assess wheelchair appropriateness (Cooper, Cooper & Boninger 2008; Hoenig, Giacobbi & Levy 2007; Horn & Gassaway 2007). Outcome measures provide information useful to wheelchair users, therapists, service providers, designers and manufacturers (Cooper et al. 2008). Evidence-based practice is also necessary for the effective use of funds, something that is especially crucial in LMIC (Cooper et al. 2008; Mortenson, Miller & Auger 2008).

Evidence-based practice can involve the appropriate application of individual knowledge of professional experts (Karthikeyan & Paris 2010). Thus, clinical judgement about the interaction between a wheelchair and its user can inform evidence-based practice. In this article, the term ‘interface’ refers to all user–wheelchair interaction. The need for evidence based on clinical judgements in no way minimises the widely recognised importance of patient report outcomes (Burke et al. 2008). However, while patient report measures are based on the experience of wheelchair users, professional report measures are informed by clinical judgement developed from training and experience. Unlike most wheelchair users, wheelchair providers are familiar with a wide range of wheelchair options (Batavia 2010). Wheelchair service providers with depth of wheelchair experience have the broadest base of experience for the assessment of the user–wheelchair interface. This experience informs individual clinical assessment (World Health Organization 2008) and can also inform the development of a brief measure intended for preliminary data or for tracking broad patterns in larger populations. Although based on clinical judgement, the best measure would also reflect an individual user’s experience.

Several characteristics of questionnaires must be considered in the development of a measure proposed to meet these needs. For some questionnaires it is not clear if they are meant to be completed by wheelchair providers or wheelchair users (Kumar et al. 2013; Schmeler et al. 2017). It is important that a questionnaire give information about who should complete it, as well as describe the target audience and purpose of the data collected (Horn & Gassaway 2007; Williams 2003). In addition to lack of specificity to participant population, a lack of specific focus on target information resulting in low discriminatory validity is also a challenge of many questionnaires (Hoenig et al. 2007; May 1997). Some measures are applied to many types of assistive technology (Demers, Weiss-Lambrou & Ska 2002) and as such offer little data on the impact of specific parts of the users’ wheelchair (May 1997). Many wheelchair outcomes are aimed at assessing the users’ ability or quality of life rather than the users’ interface with their wheelchair. As might be expected, these are very sensitive to differences in wheelchair users’ capacity, resulting in any information on the interface being overwhelmed by wide differences in users’ capacity (Kirby et al. 2004; Mills 2003; Mortenson, Miller & Miller-Pogar 2007; Rushton et al. 2011; Stanley et al. 2003). Other tools assess multiple factors together, such as maintenance condition and appropriateness, or combine multiple components into one score (Karmarkar, Collins & Cooper 2009) and consequently have low resolution for data that could result in responsive changes in the design of a specific wheelchair component (Rispin et al. 2017b). To be useful for wheelchair modification and design changes, a tool must also have high discriminatory validity (Jerosch-Herold 2005; May 1997). This means that data should not be grouped in domains in such a way that individual factors become obscured (Williams 2003). Numerical data suitable for parametric statistical analysis, such as data produced by visual analogue score responses, has been shown to increase discriminatory validity (Rispin et al. 2013; Rispin, Tutt & Sosa-Saenze 2016; Walpole et al. 2006; Wewers & Lowe 1990). In addition, a mixed-methods tool that yields both quantitative and qualitative data also increases discriminatory ability, because it provides qualitative explanations, which can be categorised to explain numerical results (Fielding 2012). For example, instead of merely reporting low scores for transferring into and out of a specific wheelchair type, a mixed-methods tool would also report comments, which might refer to armrests, chair height, brakes and so on. The repetition of a specific part that hinders transfers in the comments would allow researchers to discriminate between parts of the wheelchair that are functioning well and those that are not.

For utility in clinical practice and field studies, an outcome measure must be as brief as possible. The length of research questionnaires has an impact on administrative burden, and thus usefulness, for busy professionals (Burns et al. 2008). There exists a tension between brevity and collection of comprehensive data. However, a questionnaire that is too long for general use will yield little useful data in practice. In addition, tracking clients over time is challenging in LMIC (Rispin et al. 2017a), so cross-sectional data collection is more likely to be used than data collected over time. While an instrument appropriate for cross-sectional data collection may also be used over time, it is important that it be able to be used at a single time as well. A professional report tool that is based only on professional opinion could be employed even when wheelchair users and caregivers only speak a local language not held in common with wheelchair professionals, as may occur in LMIC. English is becoming a key global language of higher education and Internet communication with the result that an outcomes tool written in globally accessible English has the broadest scope of use internationally (Park & Wee 2017). Thus, while there is a need for such a questionnaire to have precise wording, there is also a need for simple wording that is easy to understand and translate. Finally, a tool intended for use in LMIC would logically focus on wheelchair types present there, and almost all wheelchairs encountered in most LMIC are manual wheelchairs (Pearlman et al. 2009).

The Wheelchair Interface Questionnaire (WIQ) is being developed to meet the need for a tool to yield highly discriminatory data based on a professional report snapshot of the interface between a user and his or her wheelchair. Our hypothesis was that repeated survey rounds with wheelchair professionals with international experience would support the face and content validity of the WIQ.

## Methods

### Questionnaire development

The Wheels Project is an interdisciplinary undergraduate research programme started at LeTourneau University in 2010. The Wheels Project partners with a school for children with disabilities in Thika, Kenya, to conduct field studies. The goal of these field studies is to provide manufacturers with data that can spark responsive design changes: changes to wheelchair design based on their function in a real-world environment. In addition to data on durability, user satisfaction and ease of rolling for different wheelchair types, we sought an outcome measure that would provide professional report data specifically on the quality of the interface between a user and his or her wheelchair. Over several years, we collaborated with other wheelchair researchers and conducted informal searches of the available literature seeking to find an outcome measure that would work for this purpose in our studies in Kenya. We attempted to use the Wheelchair Assessment Checklist. However, much of the data concerned wheelchair maintenance condition, and the questions to do with the user–wheelchair interface were categorised and analysed in a way that merged the two data types and merged components (Karmarkar et al. 2009). We then tried to use a simple modification of the Wheelchair Components Questionnaire (Wee & Rispin 2015). However, we found that while this gave distinct information on components, it did not include some key aspects of the interface between a user and a wheelchair.

Development of the WIQ began with a rough draft composed by the research team, informed by experience gained through these earlier studies. The focus of the WIQ was to be solely the interface between users and their wheelchairs. It was not to directly address any other aspect of quality of life. Questions were designed to be specific enough to isolate problems and inform responsive change. To address the need for high discriminatory validity, questions utilised a visual analogue scale format to provide numerical data suitable for parametric statistical analysis, with accompanying comments providing explanatory qualitative data. Like the Wheelchair Components Questionnaire, the WIQ retained questions regarding regions of the wheelchair corresponding to regions of a user’s body. Additional questions were added to the initial draft to assess other aspects of the interface. Because of heterogeneity in the capacity of wheelchair users, these questions had to be worded very carefully to keep a level playing field for all wheelchair users. For example, using the WIQ, mobility should be rated comparatively to the maximum mobility possible for a particular user. This way, an appropriate interface would not receive a low score because its user has very limited mobility. There was a commitment that the questionnaire would be brief and that the language would be clear to those speaking English as a second language.

To avoid difficulties in tracking wheelchair users over time, this tool was to be a snapshot of the quality of the interface between a wheelchair user and his or her wheelchair. To avoid loss of data when a wheelchair user was non-verbal or spoke a different language than the researcher, the WIQ was designed to be completed without verbal interaction with the wheelchair user. In other words, the WIQ did not include a formal interview process and could be completed using solely the rater’s informed clinical opinion based on visual and tactile observation. However, if verbal interaction with the wheelchair user or caregiver was possible, the assessor may choose to broaden their observations to include information obtained from the wheelchair user or caregiver. The assessor’s clinical judgement may sometimes be influenced by these interactions. Similarly, the WIQ does not require the user to perform movements, so raters can assess an interface without communicating actions to the wheelchair user. However, the accuracy of a rating would be improved if the wheelchair user did perform some movements for the rater, so communication is encouraged when possible. The target audience for data resulting from the use of the WIQ was initially wheelchair manufacturers addressing design issues. For example, if wheelchair providers repeatedly give low scores for many users regarding the ease of transfer into and out of a certain type of wheelchair, designers might consider modifications to address that low rating. However, as development continued, the target audience broadened to include service providers in clinical settings.

### Methodology for validity study

Any tool used in clinical research must be valid (Karmarkar et al. 2009). Validity is the degree to which a test measures what it is intended to measure (Williams 2003). Face validity, which is considered the initial form of validity, considers whether the tool appears valid to the population qualified to utilise the tool. Content validity takes into account both comprehensiveness and representativeness of the content of a tool (Yaghmale 2009 2003). Content validity is assessed by professional judgement and is improved by the inclusion of at least five experts (Yaghmale 2009). Informed opinion that approaches consensus from multiple experts during the development of a tool indicates the face and content validity of that tool (Burns et al. 2008; Williams 2003; Yaghmale 2009). One method for reaching consensus among experts is called the Delphi method, which involves the conduction of several rounds of a survey, utilising feedback to adjust after each round (Brady 2015). If participants involved have a similar background, only 10–15 participants are needed, and fewer than three rounds may be acceptable for a study (Hsu & Sandford 2007). A Delphi-style survey of wheelchair experts was planned to investigate the face and content validity of the WIQ. At least two Delphi rounds would be included, with the option to continue the survey format or conduct a less traditional focus group.

### Participant characteristics

Because wheelchair service providers would be completing the questionnaire, a cadre of service providers was sought as study participants to assist with the development, face validity study and content validity study of the WIQ. A range of service providers was desired, with a majority of occupational therapists and physical therapists. Clinicians known to the researchers and met through professional contacts were invited to participate in the study. Although the WIQ was developed for use in Kenya, it was also intended to be used in other LMIC around the world, so service providers with international wheelchair experience were approached. International experience, defined for this study as experience outside of Europe and North America, was self-reported by participants. For the online surveys, exclusion criteria included less than 5 years of experience, no global wheelchair experience or no measurable certifications or qualifications.

### Ethical considerations

The study design was approved by the Institutional Review Board at LeTourneau University in an approval letter (protocol number 1703001174, reference: Biology Department of LeTourneau University).

The first stage of the study included a two-round online survey, loosely based on a Delphi study. A snowball sampling method was used, with those who had initially joined the study recommending others. The survey rounds were followed by an in-person focus group in Kenya for validation expressly in the Kenyan context.

### Study design

Participants in all three rounds were given a copy of the latest draft of the WIQ along with background information about the purpose and focus of the WIQ. They then completed a survey, which enabled them to respond to the purpose of the questionnaire, rate the current draft, provide feedback and make recommendations about what would be important to include in such a tool. A total of 17 of the wheelchair professionals invited participated in the two surveys. Surveys were conducted using LimeSurvey version 2.5, an open source application that allowed researchers to develop an original survey, send it to participants and collect responses for analysis. The online format was beneficial because it was accessible to participants anywhere around the world. In addition, responses could be anonymous, allowing for a wide range of opinions to be voiced. Changes were made to the WIQ in response to the first survey round, and an updated draft of the WIQ and LimeSurvey response survey was sent to the participants involved in Round 1 as well as newly invited participants joining the study for the first time. Questions on the LimeSurvey asked participants to rate and comment on the value of and need for the WIQ; the title, format and usefulness; and to rate and respond to individual questions on the questionnaire. Each aspect of the questionnaire and each question was rated on a seven-point Likert scale, with a space for comments. After each round, the ratings were analysed, and the comments were categorised by topic. Questions that received two or more ratings below four on the seven-point scale were changed or deleted. Repeated and important comments were considered in editing each draft of the questionnaire.

After the two online survey rounds, a focus group was conducted in Kenya. Kenya was chosen as a representative low-income country because the researchers were conducting ongoing studies in Kenya and knew there would be a group of wheelchair professionals with broad experience in LMIC present. Nine of these professionals were chosen by convenience sampling to participate in the study. Each participant used the most recent draft of the WIQ to rate the interface between a wheelchair user and his or her chair. Two of the participants used that draft of the WIQ to evaluate more than 20 wheelchair users and their wheelchairs. Fifty WIQs were completed in total. This hands-on experience gave participants insight and understanding of the WIQ. Participants then rated the WIQ on a paper version of the online survey that had been used in the previous two rounds of the study. Subsequently, there was open discussion, which was recorded by a research assistant. Written comments from the paper version of the survey along with comments from the open discussion were categorised and responded to, both in person during the focus group study and during editing of the WIQ.

## Results

Because our participants were recruited using snowball sampling, more participants were added throughout the study. Eight participants with global wheelchair experience completed the first round of the survey, with nine additional participants in the second round. Table 1 shows qualifications and years of wheelchair experience for each of the participants. Participants included occupational therapists, physical therapists, seating specialists and medical doctors with experience in rehabilitation and wheelchair provision. Participants in the Kenya focus group included occupational therapists, physical therapists, orthopaedic technologists, wheelchair technicians and one educator with extensive experience working with students in wheelchairs. Two of the focus group participants had been a part of the online survey.

**TABLE 1:** Study participants for each stage of research (*n* = 24).†

Qualifications or training	Years of wheelchair experience	Years of global wheelchair experience
**Participated in Rounds 1 and 2 (*n* = 8)**		
MPT	6	6
MOT, ATP	7	1
MPT, ATP, SMS	10	8
MPT	15	2
DPT	17	10
MD in rehabilitative medicine	20	14
CRTS, ATP	25	15
PTA, ATP	33	4
**Participated in Round 2 only (*n* = 9)**		
DPT	7	5
Diploma	9	5
DPT, ATP	15	6
MOT, WSTP-I	15	11
MPT	20	20
MOT, ATP	21	6
DPT, PHD	30	9
MOT, ATP, SMS	30	14
OTR, SMS, ATP	40	11
**Participated in focus group (*n* = 9)**		
Wheelchair technician, WSTP-B	1	1
Orthopaedic technologist, WSTP-B	5	5
MOT, ATP	7	1
MPT	7	7
DPT	8	8
OT Cert, WSTP-I	8	8
OT Cert, WSTP-I	8	8
Educator	20	20
MPT, ATP	37	1

MPT, Master of Physical Therapy; MOT, Master of Occupational Therapy; ATP, assistive technology professional; SMS, seating mobility specialist certified by the Rehabilitation Engineering and Assistive Technology Society of North America; DPT, Doctor of Physical Therapy; CRTS, certified rehabilitation technology supplier; PTA, physical therapy assistant; WSTP-I/WSTP-B, Wheelchair Service Training Package – Intermediate or Basic Level by the World Health Organization; OTR, registered occupational therapist.

† , two focus group participants also participated in the online survey.

Table 2 shows mean Likert scores for aspects of the WIQ and topics of the questions included in the final version of the questionnaire. The table is divided by rounds of the online surveys and the focus group in Kenya. As the table shows, the average scores for all final questions were above four on a seven-point scale.

**TABLE 2:** Mean Likert scores from Delphi Round 1, Round 2 and focus group.

Variable	Round 1 Mean (SD) *r* = range	Round 2 Mean (SD) *r* = range	Focus group Mean (SD) *r* = range
**Aspect of questionnaire**			
A brief questionnaire asking directly about the interface between user and wheelchair	6.5 (1.0) *r* = 4–7	6.6 (0.8) *r* = 5–7	6.1 (1.0) *r* = 4–7
Usefulness in large field studies	6.1 (1.0) *r* = 4–7	6.4 (1.2) *r* = 4–7	N/A†
Intention to be inclusive of most manual wheelchairs and their users	6.0 (1.4) r = 2–7	6.3 (1.3) r = 2–7	6.2 (0.9) r = 4–7
Based on informed professional opinion	5.0 (1.7) *r* = 2–7	5.3 (1.6) *r* = 2–7	5.4 (1.7) *r* = 4–7
Current title	N/A	4.5 (1.8) *r* = 1–7	5.7 (1.6) *r* = 5–7
Format	6.3 (1.1) *r* = 4–7	6.1 (1.4) *r* = 2–7	6.2 (0.5) *r* = 6–7
Information gathered at head	5.7 (1.0) *r* = 5–7	5.4 (1.5) *r* = 2–7	6.3 (0.5) *r* = 6–7
Instructions	N/A	5.4 (1.9) *r* = 1–7	5.8 (1.9) *r* = 1–7
Questionnaire overall	5.5 (1.3) *r* = 3–7	5.3 (1.8) *r* = 1–7	6.1 (0.6) *r* = 5–7
**Topics of questions**			
Fit of this wheelchair for this user	6.3 (1.0) *r* = 5–7	6.4 (1.3) *r* = 3–7	6.4 (0.9) *r* = 5–7
Wheelchair’s facilitation of mobility surfaces and obstacles commonly encountered	5.0 (2.0) *r* = 2–7	6.1 (1.4) *r* = 3–7	6.7 (0.7) *r* = 5–7
Wheelchair’s facilitation of mobility in small spaces	5.5 (1.9) *r* = 2–7	5.8 (1.6) *r* = 3–7	6.9 (0.3) *r* = 6–7
Wheelchair’s facilitation of toilet activities	5.2 (2.2) *r* = 1–7	N/A	N/A
The ease of bringing this wheelchair on a car or van	5.4 (1.8) *r* = 2–7	5.5 (1.9) *r* = 2–7	6.7 (0.5) *r* = 6–7
Wheelchair’s prevention of pain or harm to:			
Upper limbs	5.6 (1.8) *r* = 2–7	5.3 (1.6) *r* = 2–7	5.9 (1.5) *r* = 3–7
Trunk and head	5.5 (2.0) *r* = 1–7	5.3 (1.3) *r* = 3–7	6.3 (1.0) *r* = 4–7
Proximal lower limb	5.5 (1.6) *r* = 3–7	5.4 (1.7) *r* = 1–7	6.3 (1.0) *r* = 4–7
Distal lower limb	5.5 (2.0) *r* = 1–7	5.5 (1.6) *r* = 1–7	6.3 (0.5) *r* = 6–7
Wheelchair’s postural support	N/A	5.9 (1.5) *r* = 3–7	6.4 (0.7) *r* = 5–7
Wheelchair’s facilitation of desk or table activities	N/A	5.8 (1.3) *r* = 4–7	6.5 (0.5) *r* = 6–7
Wheelchair’s facilitation of sitting up to make eye contact for social interaction	6.0 (1.6) *r* = 3–7	5.7 (1.4) *r* = 3–7	6.4 (0.7) *r* = 5–7
Wheelchair’s facilitation of transfers	N/A	N/A	N/A

Note: Rated on a seven-point scale from 1 (‘serious problems or omissions’) to 7 (‘looks functional and appropriate’).

† , Some questions were not asked in all three surveys, either because the focus of the survey was different or because questions were not yet added or omitted.

SD, standard deviation; N/A, not applicable.

Table 3 shows the participants’ comments from the online survey, categorised by topic. Both Likert scale scores and comments indicated consensus on the need for a questionnaire focused on the user–wheelchair interface and completed by service providers. Participants used comments to indicate what they felt would be important to include in such a tool. There was consensus that the focus on manual wheelchairs was appropriate for use in LMIC. Participants supported the idea that the WIQ would be useful in field studies to provide data to manufacturers that would enable design change. They felt it could also be useful in clinical practice as an initial overview before a more detailed clinical assessment. Comments about the demographic information collected at the top of the questionnaire resulted in the inclusion of a five-point scale for upper body strength and for trunk and head control. Comments strongly emphasised the importance of clear and simple vocabulary easily understood by second language English speakers. It was thought that in the future this would also facilitate translation of the WIQ. Several study participants emphasised the importance of brevity. There was an emphasis on the importance of a tight focus on the wheelchair interface. The importance of grouping body areas according to the impact of different wheelchair regions was discussed to allow for meaningful responsive change in design and fitting. Several participants offered alternative wording of questions on the WIQ.

**TABLE 3:** The frequency of comments by topic.

Topic	Frequency in Round 1	Frequency in Round 2
Affirm value of proposed questionnaire	6	7
Input on language and formatting	5	9
Discussion about user inclusion	5	7
Affirm need for brevity	3	2
Input on wording of questions	16	8
Need for postural support question	3	Added after Round 1
Eliminate toilet question	5	Deleted after Round 1
Condense pain and harm questions	1	2

In responding to participant comments, questions were simplified, clarified and shortened. The question regarding pain was divided by body region. Several participants suggested that a question regarding toilet activities should be deleted because for many wheelchair users it would not be directly impacted by the user–wheelchair interface, or the factors that were impacted were already addressed in a question about mobility in small spaces. Because this question had low mean ratings, it was deleted. Participants also suggested adding a question regarding postural support and another regarding the facilitation of desk and table activities.

The focus group in Kenya again emphasised the importance of brevity. Consensus was reached that the subtitle should include the term ‘service provider’. It was suggested that service providers mark questions ‘N/A’ for questions that do not apply to the interface they are rating to discriminate between questions that truly do not apply and questions forgotten by a rater. This would minimise the possibility of missing data. Wording for the question regarding the wheelchair’s facilitation of social interaction was discussed and modified. There was consensus that a question regarding a wheelchair’s facilitation or hindering of transfers should be included. This grew out of the assessment of one type of wheelchair. Those with extensive experience with that wheelchair type knew that it consistently hindered transfers and wanted an avenue to provide feedback to manufacturers. The group agreed this was an important part of a user’s interface with his or her wheelchair and came to consensus on wording for this additional question. There was consensus on the value of a questionnaire that can be administered with or without interaction with the wheelchair user. This would enable the WIQ to be useful when a wheelchair user is a child, non-verbal adult or a speaker of a different language. However, there was also consensus on encouraging wheelchair providers to include auditory information from the users and caregivers whenever possible to broaden their frame of reference while rating a wheelchair.

**FIGURE 1:**
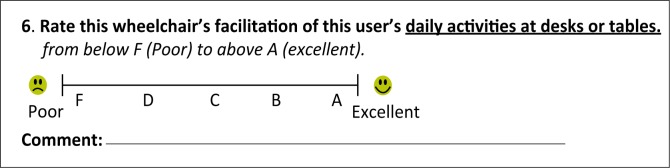
An example of a question on the Wheelchair Interface Questionnaire.

After modification and refinement following each round of the study, the WIQ now has nine questions. The first question regarding pain has sub-questions regarding four body regions that are to be analysed as individual questions. Box 1 shows the wording of the questions on the WIQ. Each question is answered using a visual analogue scale format with an accompanying comment, to yield both qualitative and quantitative data. Figure 1 is an example of a question on the WIQ.

Box 1 The wording of the questions included in the most recent version of the questionnaire.

- Rate how well this wheelchair prevents pain or harm to …
  ▪ this user’s head and trunk
  ▪ this user’s shoulders, arms, and hands
  ▪ this user’s hips, buttocks, and thighs
  ▪ this user’s calves, ankles, and feet.
- Rate the dimensions of this wheelchair for this user.
- Rate this wheelchair’s postural support for this user.
- Rate this wheelchair’s facilitation of mobility across all surfaces and obstacles this user is likely to encounter in daily life.
- Rate this wheelchair’s facilitation of mobility in small spaces.
- Rate this wheelchair’s facilitation of this user’s daily activities at desks or tables.
- Rate this wheelchair’s facilitation of social contact for this user.
- Rate the ease of transporting this wheelchair in/on a car, van, or other means of transport this user is likely to encounter.
- Rate the ease of transferring in and out of this wheelchair for this user, with or without the help of an assistant.

Note: All questions included the phrase ‘from below F (poor) to above A (excellent)’.

## Discussion

### Discussion of results

Input from 24 participants with over 300 combined years of wheelchair experience confirmed and informed the face and content validity of the WIQ and each of its questions. Focus group members’ hands-on experience using the questionnaire informed their feedback about the questionnaire’s content. Our results support the hypothesis that the WIQ has initial face and content validity. With additional reliability and validity testing, it can become a useful tool for assessing the user–wheelchair interface in LMIC.

Agreement among study participants confirmed our hypothesis that a tool to yield professional opinion on the quality of the interface between a wheelchair and user would indeed be useful. There was also agreement that the lack of a standardised level of verbal interaction with a wheelchair user or caregiver would greatly increase the WIQ’s utility and applicability in large studies. The ability to complete the questionnaire without interviewing users allows service providers to assess the wheelchair interface of users who cannot be interviewed. In LMIC, wheelchair providers may not always be able to communicate easily with wheelchair users or their caregivers because of language barriers. In other cases, wheelchair users who are non-verbal or very young may be in an institutional or boarding school situation without a long-term established personal caregiver. Feedback from the focus group confirmed these considerations. However, participants felt that any feedback possible should be considered as part of the observational data informing the wheelchair provider’s professional opinion as expressed through the WIQ.

The fact that the WIQ is intended to be a brief snapshot of the interface between a user and his or her wheelchair at a given moment in time also broadens the venues in which it may be used. At the same time, because the WIQ is a brief questionnaire based solely on a professional opinion of the interface at a given moment in time, it is necessarily much less complete than a clinical relationship with repeated clinical assessments and records kept over a client’s lifespan. While there was feedback that the WIQ might be of clinical use, it would not in any way replace the need for a full assessment or clinical records. Instead, the WIQ could function as an initial indication of what assessment might be needed. Some participants suggested that if thresholds for overall score were established, it could be used as an evidence-based indication of the need for a new chair or a modified chair.

Repeated confirmation that a brief questionnaire using simple language was more likely to be utilised by busy providers reminded researchers to keep questions brief and simple. This facilitates quick comprehension and completion for those who may speak English as a second language, as well as easier translation. Because the WIQ is intended as a professional report tool, and most wheelchair providers have had some variety of post-secondary education, it is likely that the WIQ may need only to be translated into the languages used for higher education.

Unlike measures focused on the quality of life, mobility level, skills or confidence levels of wheelchair users, the WIQ is designed to avoid rating the wheelchair users’ capability level. This was a challenge because it required that questions be written to enable a level playing field for all users. Because the WIQ is intended only for manual wheelchairs, this means that each interface is compared to the assessor’s understanding of the best possible manual wheelchair interface for that user. Yet there will be users who would have benefitted from a power wheelchair. Currently, this is not reflected in the results from the WIQ. In the future, as the global situation changes, a version of the WIQ could be developed to include the option of power wheelchairs.

Participants confirmed the value of the inclusion of questions about specific wheelchair regions. The wheelchair was divided by region, supporting different body parts, rather than by part to avoid loss of data when wheelchairs do not all have the same part: for example, questions ask about postural support instead of about backrests and armrests because a wheelchair may not have an armrest. This division also keeps the questionnaire focused on the wheelchair user. It is interesting that participants wanted to group the questions regarding pain or discomfort into one question but keep aspects of the wheelchair interface divided by body part. This was to enable quicker clinical response to ameliorate problems leading to pain or damage. The WIQ may also enable specific feedback to wheelchair manufacturers if there are repeated characteristic problems with a certain wheelchair type.

### Limitations and future work

A greater number of participants would have provided more feedback. Although some study participants had international experience in South America, Africa and Asia, most were from North America and Kenya. The inclusion of wheelchair providers familiar with global wheelchair work delivers a broad framework of experience. However, further studies that include Asian and South American wheelchair providers would be beneficial. The final focus group in Kenya gave an additional level of validation, but limited time in the earlier online survey resulted in only two rounds, diverging from a traditional three-round Delphi-style survey.

Additional validity testing remains to be done. The time to complete the WIQ was not formally tracked, and this needs to be done in a systematic way to confirm that the WIQ is brief enough to be useful in many settings. Inter-rater reliability testing is planned as this is the method most commonly utilised to assess the reliability aspect of validity. It is done by comparing the scores of a group of assessors rating the same subjects. Construct validity compares results from one measure to another somewhat similar measure to see if they move together as expected. A study of construct validity is also planned. Test–retest reliability and other tests remain to be done as well.

The WIQ itself is necessarily limited by a rater’s knowledge, experience, biases and training. Raters’ knowledge is not only limited by their qualifications and experience but also by the level of communication they can have with the user. An important strength of the WIQ is that it does not require a standardised level of communication. However, communication adds to a rater’s depth of understanding, so the variance of communication ability among raters will have at least some impact on their ratings. Because of this intrinsic limitation, the results of the planned inter-rater reliability study are of interest.

Discriminatory validity is the ability of a measure to discern meaningful difference in a sensitive manner. Study participants confirmed the importance of qualitative and quantitative data, as well as that of data that would provide information specific enough to enable responsive modification and design changes. In other questionnaires using a similar format, the visual analogue scale provides continuous quantitative data while the comments provide qualitative data that gives a reason behind the rating (Rispin et al. 2013; Rispin et al. 2017b). High discriminatory validity for the WIQ cannot be confirmed until a large study with multiple sets of 10 or more individuals in different types of wheelchairs is completed. This is planned to confirm that the WIQ is able to identify repeated patterns in specific chair types. For example, the WIQ should be able to identify a design issue that hinders transfers for most users.

## Conclusion

This study supports the face and content validity of the WIQ as a measure focused specifically on obtaining professional report data on the interface between a wheelchair user and his or her wheelchair at a specific moment in time. Two rounds of a survey of expert opinion and one focus group supported face and content validity and informed the final draft of the questionnaire. The WIQ is intended to be used by wheelchair providers with a background that enables informed clinical judgement. Questions are designed to identify specific problems with the user–wheelchair interface. When further validation is completed, the WIQ could be used in large field studies to provide data that facilitates responsive design changes by manufacturers. In a clinical setting, the WIQ could indicate problem areas, which could be investigated further in a more detailed clinical assessment.
